# Characterization of monoclonal antibodies that specifically differentiate field isolates from vaccine strains of classical swine fever virus

**DOI:** 10.3389/fimmu.2022.930631

**Published:** 2022-07-19

**Authors:** Shijiang Mi, Lihua Wang, Hongwei Li, Fei Bao, Rachel Madera, Xiju Shi, Liying Zhang, Yingying Mao, Renhe Yan, Xianzhu Xia, Wenjie Gong, Jishu Shi, Changchun Tu

**Affiliations:** ^1^ State Key Laboratory for Zoonotic Diseases, Key Laboratory for Zoonoses Research of the Ministry of Education, College of Veterinary Medicine, Jilin University, Changchun, China; ^2^ Changchun Veterinary Research Institute, Chinese Academy of Agricultural Sciences, Changchun, China; ^3^ Department of Anatomy and Physiology, College of Veterinary Medicine, Kansas State University, Manhattan, KS, United States; ^4^ School of Biotechnology, Southern Medical University, Guangzhou, China; ^5^ Institute of Animal Qurantine Reserach, Science and Technology Research Center of China Customs, Beijing, China; ^6^ College of Animal Science and Technology, Jilin University, Changchun, China; ^7^ Department of Research & Development, Guangzhou Bioneeds Biotechnology Co., Ltd, Guangzhou, China; ^8^ Jiangsu Co-Innovation Center for Prevention and Control of Important Animal Infectious Diseases and Zoonoses, Yangzhou University, Yangzhou, China

**Keywords:** CSFV, E2, E^rns^, differentiating mAb, characterization, epitope

## Abstract

Classical swine fever virus (CSFV) is a major animal pathogen threatening the global pork industry. To date, numerous anti-CSFV monoclonal antibodies (mAbs) and their recognizing epitopes have been reported. However, few mAbs were systematically characterized for the capacity to differentiate field CSFV isolates from CSF vaccine strains, and the molecular basis associated with antigenic differences between vaccines and field isolates is still largely unknown. In the present study, recombinant CSFV structural glycoproteins E2 of both virulent and vaccine strains and E^rns^ of vaccine strain were expressed using eukaryotic cells and murine mAbs generated against E2 and E^rns^. After serial screening and cloning of the hybridomas, the viral spectra of mAbs were respectively determined by indirect fluorescent antibody assay (IFA) using 108 CSFVs, followed by Western blot analysis using expressed glycoproteins of all CSFV sub-genotypes including vaccine strains. The antigenic structures recognized by these mAbs were characterized by epitope mapping using truncated, chimeric, and site-directed mutated E2 and E^rns^ proteins. We have identified two vaccine-specific, one field isolate-specific, and two universal CSFV-specific mAbs and five novel conformational epitopes with critical amino acid (aa) motifs that are associated with these five mAbs: ^213^EPD^215^, ^271^RXGP^274^, and ^37^LXLNDG^42^ on E2 and ^38^CKGVP^42^, W^81^, and D^100^/V^107^ on E^rns^. Particularly, E^213^ of E2 is field isolate-specific, while N^40^ of E2 and D^100^/V^107^ of E^rns^ are vaccine strain-specific. Results from our study further indicate that N^40^D of E2 mutation in field strains was likely produced under positive selection associated with long-term mass vaccination, leading to CSFV evasion of host immune response. Taking together, this study provides new insights into the antigenic structure of CSFV E2 and E^rns^ and the differentiating mAbs will contribute to the development of a diagnostic strategy to differentiate C-strain vaccination from natural infection (DIVA) of CSFV in terms of elimination of CSF in China.

## 1 Introduction

Classical swine fever (CSF) is a severe swine infectious disease caused by CSF virus (CSFV), which significantly impairs the pig industry worldwide. CSFV belongs to the genus *Pestivirus* within the family *Flaviviridae* together with bovine viral diarrhea virus (BVDV), border disease virus (BDV), and other newly identified pestiviruses (1, 2). The CSFV genome is a single-strand positive-sense RNA with 12.3 kb in size, which consists of a large open reading frame (ORF) and untranslated regions at both 5′ and 3′ ends (5′UTR and 3′UTR). The ORF encodes a polyprotein of 3,898 amino acids, which is processed to four structural proteins (Core, E^rns^, E1, and E2) and eight non-structural proteins (N^pro^, p7, NS2, NS3, NS4A, NS4B, NS5A, and NS5B) by host-cellular and viral proteases (2–5). Although CSFV has only one serotype, it is genetically variable and has evolved into 3 genotypes, 11 sub-genotypes based on phylogenetic analysis with its E2, 5′UTR, or NS5B gene sequences (6, 7). Changes in CSFV structural protein genes during evolution have resulted in broad antigenic differences among the field isolates as identified by serial monoclonal antibodies (mAbs) against E2 and E^rns^ (8). These two proteins are structural glycoproteins on the CSFV virion surface and directly exposed to immune cells (9). Glycoprotein E2 is the major envelope protein, and both E2 and E^rns^ can induce neutralizing antibody responses during CSFV infections (10–12). E2 has four antigenic domains (A, B, C, and D) located in its N-terminal half (13), which constitute two antigenic units, B/C (residues 1–90 aa) and D/A (residues 91–170 aa) (9, 14). Both glycoproteins also contain virulent determinants (15–17) and are responsible for virus attachment to target cells and receptor binding to mediate viral entry into target cells to accomplish the viral replication cycle (18).

CSFV is a major animal pathogen that poses a significant threat to the global pork industry. The hog cholera lapinized virus (HCLV, also called C-strain) is widely used to prevent and control CSF in China and other countries because of its high efficacy and safety profile. However, little is known about the antigenic difference between HCLV and field CSFV isolates, and it is difficult for traditional serological assays to distinguish antibody response induced by field strain infection from that induced by vaccination of live vaccine (19).

Numerous mAbs against CSFV have been generated in the last few decades for illustrating their protein functions and antigenic structures, as well as for diagnosis of the disease (13, 20–22). Most of these mAbs are directed to E2 and used to identify many linear and conformational antigenic epitopes or critical aa sites on this protein (9). Significant progress has been achieved in recent years on the generation of anti-E^rns^ mAbs and epitope mapping of E^rns^ (8, 23–25). E2 and E^rns^-specific mAbs were used to distinguish CSFV isolates based on the patterns of their reactivity with these mAbs (8, 26–28). However, there has not been any mAb showing reliable specificity of differentiating field isolates from vaccine strains, and vaccine-specific or field isolate-specific epitopes or aa motifs have not been clearly defined.

Several mAbs showed potential capacity to differentiate field isolates from vaccine strains (8, 27, 29), but only a limited number of field and vaccine viruses were used to evaluate their specificity and capability, and a systematic validation using a broad spectrum of different sub-genotypes of field isolates and vaccine strains is still needed. Moreover, the epitopes or aa motifs of vaccine strains and field isolates recognized by these differentiating mAbs have not been mapped.

Here we report the generation and systematic characterization of novel mAbs that could specifically differentiate field isolates from vaccine strains. Epitope mapping of E2 and E^rns^ glycoproteins using these mAbs shed light on how these vaccine- and field isolate-specific aa motifs contribute to the differentiation. This study obtained has significantly increased our understanding of CSFV antigenic structures, and the differentiating mAbs will facilitate to the development of DIVA assay for C-strain vaccination.

## 2 Materials and methods

### 2.1 Cell, virus, and gene syntheses

PK-15 and MDBK cell lines were stored in our laboratory and cultured in MEM or DMEM (Corning, Tewksbury, MA, USA) supplemented with 100 μg/ml penicillin, 100 IU/ml streptomycin sulfate (Gibco, Grand Island, NY, USA), and 10% fetal bovine serum (FBS) (Corning, USA) at 37°C and 5% CO_2_. Insect cell lines Sf9 and High Five (Life Sciences) used for the expression of CSFV E2 and E^rns^ proteins were cultured at 27°C in Grace’s Insect medium and Sf-900™ II SFM (1×) (Gibco, USA) supplemented with 100 μg/ml penicillin, 100 IU/ml streptomycin sulfate (Gibco, USA), and 5% FBS. Highly virulent CSFV reference SM, HCLV strain, and 106 CSFV field isolates (sub-genotypes 1.1, 2.1, 2.2, and 2.3) and bovine viral diarrheic virus 1 (BVDV1) strain 32 were stored in our laboratory. These field isolates were collected between 1990 and 2017 from 23 provinces of China (30–32) and adapted in PK-15 cells following isolation (31, 33). The growth and replication of CSFV in PK-15 cells were confirmed by indirect fluorescent antibody assay (IFA) using CSFV-specific mAb WH303 (34) as the primary antibody and Alexa Fluor 488 donkey anti-mouse IgG (H+L) (Life Technologies, MA, USA) as the secondary antibody (33). Full E2 and E^rns^ genes of various CSFV strains were synthesized at Jilin Comate Biotech Company (Changchun, Jilin, China).

### 2.2 Amplification and sequence analysis of CSFV E^rns^ and E2 genes

Viral RNA of CSFV was extracted using TIANamp Virus RNA Kit (Tiangen, China) and subsequently reverse-transcribed with SuperScript™ III First-strand Synthesis System (Invitrogen, Carlsbad, CA, USA) to prepare the first cDNA strand according to the manufacturer’s instructions. Amplification of full-length E2 and E^rns^ genes was performed using Phusion High-Fidelity DNA Polymerase (NEB, Ipswich, MA) with the above cDNA as the template and specific primers. Fifty microliters of PCR reaction contained 10 µl 5× Phusion HF Buffer, 1 µl dNTPs (10 mM), 2.5 µl forward primer, 2.5 µl reverse primer, 1 µl cDNA, 1.5 µl DMSO, 0.5 µl Phusion DNA polymerase, and 31 µl ddH_2_O. PCR cycling was conducted at 98°C for 30 s, followed by 35 cycles of 98°C for 10 s, 55°C for 30 s, and 72°C for 30 s, and the final extension was at 72°C for 10 min. The specific PCR amplicons were commercially sequenced with ABI sequencer 3730XL at Jilin Comate Biotech Company (Changchun, Jilin, China). Alignments of nucleotide and deduced amino acid sequences of E2 and E^rns^ were conducted using CLC Sequence Viewer 7.6.1 (Qiagen, Hilden, Germany). Phylogenetic analysis based on CSFV E2 complete genes was conducted with MEGA 7.0 to construct the phylogenetic tree. To strengthen the robustness of the results, both neighbor-joining and maximum-likelihood methods were used. The reliability of the generated trees was determined using 1,000 bootstrap replicates.

### 2.3 Expression and purification of CSFV E^rns^ and E2 proteins

Expressions of HCLV-E^rns^ and HCLV-E2 proteins were performed with the baculovirus expression system as previously described (35). In brief, amplifications of HCLV-E^rns^ and E2 genes flanked by the signal peptide sequence at the N-terminal and 6×His-tag sequence at the C-terminal were performed by PCR using the above procedure. The amplified fragment was subcloned into pFastBac 1 plasmid, and the recombinant vectors were transformed into MAX Efficiency DH10Bac competent cells maintaining Bacmid and helper plasmid (Invitrogen, USA); the transformants were then plated on LB agar plates containing 50 μg/ml kanamycin (Solarbio, Beijing, China), 7 μg/ml gentamicin (Solarbio, China), 10 μg/ml tetracycline (Solarbio, China), 100 μg/ml X-gal (TaKaRa, Dalian, China), and 40 μg/ml IPTG (Solarbio, China). After incubation at 37°C for 48 h, white colonies were cultured and the recombinant bacmid was verified by PCR. Then the recombinant bacmid DNA was extracted using partial reagents of AxyPrep™ Plasmid Miniprep Kit (Axygen, China). One microgram of recombinant bacmid DNA was subsequently transfected with Cellfectin II Reagent (Gibco, USA) into insect cells Sf9 to get recombinant baculovirus, and three passages of recombinant baculovirus stocks were prepared. The third passage of recombinant baculovirus was inoculated into High Five insect cells to express proteins at 27°C for 72 h. Recombinant proteins were purified by Ni-NTA agarose beads according to the manufacturer’s protocol (Invitrogen, USA). The purified HCLV-E^rns^ and HCLV-E2 proteins were assessed by SDS-PAGE and Western blot. The purified E2 protein of highly virulent SM strain (SM-E2) was provided by Guangzhou Bioneeds Biotechnology Co., Ltd. (Guangzhou, Guangdong, China), which was expressed with the lentivirus expression system in 293T cells.

### 2.4 Generation and isotype determination of anti-E^rns^ and anti-E2 mAbs

Generation of mAbs against purified HCLV-E^rns^ and HCLV-E2 proteins was conducted using our published methods (36). Animal experiments for production of the mAbs were approved by the Institutional Animal Care and Use Committee (IACUC#3517) at Kansas State University and conducted under strict adherence to the IACUC protocols. To generate mAbs against the SM strain, purified SM-E2 protein was mixed with equal volumes of Freund’s complete adjuvant (Sigma, St. Louis, MO) and each Balb/c mouse with 50 μg/500 μl of mixture was immunized *via* intraperitoneal injection. After a 2-week interval, the second and third booster vaccinations were performed with the same method of the first vaccination, while the adjuvant was replaced by Freund’s incomplete adjuvant (Sigma, USA). Ten days after the third booster vaccination, the mouse with the highest E2 serum antibody titer was humanely euthanized and its spleen cells were fused with SP2/0-Ag14 cells to obtain the hybridoma cells secreting anti-E2 mAbs, which were then subcloned by the limited dilution method (37). The animal protocols and procedure for production of mAbs against SM-E2 were reviewed and approved by the Animal Care and Use Committee of the Southern Medical University (protocol number L2014076). The isotype of mAbs was determined using mouse antibody-isotyping ELISA kit according to the manufacturer’s protocol (Biodragon, Beijing, China).

### 2.5 Identification of specificity and reactivity of anti-E^rns^ and anti-E2 mAbs

To test the specificity of anti-E^rns^ and anti-E2 mAbs, MDBK cells were seeded to the 96-well culture plate at a concentration of 1.0 × 10^4^ cells/well with DMEM containing 10% FBS. After overnight culture, cells were infected with BVDV-1 strain 32 and then incubated in 5% CO_2_ at 37°C for 72 h. Following incubation, the infected cells were fixed with 80% cold acetone at -20°C, followed by washing three times with PBS, then the virus was detected by IFA with the generated anti-E^rns^ and anti-E2 mAbs as primary antibodies and Alexa Fluor 488 donkey anti-mouse IgG (H+L) (Life Technologies, MA, USA) as the secondary antibody. In parallel, a specific polyclonal antibody against BVDV1-E2 protein (Bioss, Beijing, China) was used to detect the growth of BVDV1 as the positive control.

To identify the reactivity of anti-E^rns^ and anti-E2 mAbs, PK-15 cells were seeded to a 96-well culture plate at a concentration of 1.0 × 10^4^ cells/well with MEM containing 10% FBS. After overnight incubation in 5% CO_2_ at 37°C, cells were infected respectively with CSFV SM strain, HCLV, and various field isolates at a MOI of 0.1 and then incubated in 5% CO_2_ at 37°C for 72 h. The infected cells were fixed and detected by IFA described above.

To further characterize the reactivity spectrum of the mAbs, E^rns^ and E2 proteins of CSFV field isolates and vaccine strains of other sub-genotypes unavailable in our laboratory were respectively expressed in the above baculovirus system using synthesized gene fragments based on published sequences in GenBank [**Figure S1** and **Table S1** (7, 17, 38–45)]. The binding of anti-E^rns^ and anti-E2 mAbs with recombinant E^rns^ and E2 proteins was analyzed by Western blot as previously described (36, 46). Briefly, the supernatant of Sf9 cells infected by recombinant baculovirus for 72 h was collected and treated with SDS loading buffer (Bio-Rad, Shanghai, China) without reducing reagent dithiothreitol (DTT), then loaded in 5%–10% (for E2 protein) or 5%–12% (for E^rns^ protein) SDS-polyacrylamide gel. After electrophoresis, the proteins in the gels were transferred onto a nitrocellulose (NC) membrane (Cytiva, Shanghai, China) and subsequently reacted with anti-E2 and E^rns^ mAbs. In parallel, E2 and E^rns^ protein expression was confirmed by Western blot using mouse anti-His mAb (GenScript, Piscataway, NJ, China) and Alexa Fluor 680 donkey anti-mouse IgG (H+L) (Life Technologies, Framingham, MA, USA). Then the NC membranes were scanned by Odyssey Infrared Imaging System (LI-COR Biosciences, Lincoln, NE, USA) according to the manufacturer’s instruction.

### 2.6 Mapping epitopes recognized by mAbs

To identify epitope types (linear or conformational) recognized by these mAbs, recombinant E^rns^ and E2 proteins of HCLV and SM strains expressed by the baculovirus and *E. coli* expression systems described above were treated using loading buffer with or without DTT, which can alter the structure of proteins by reducing disulfide bonds, and then subjected to Western blot with anti-E^rns^ and anti-E2 mAbs prepared above.

To map the critical amino acids forming the epitope, amino acid sequences of E2 and E^rns^ proteins reacted and unreacted with the mAbs were retrieved from GenBank and the multiple-sequence alignments were conducted using CLC Sequence Viewer 7.6.1 as described above to identify the candidate residues for site-directed mutagenesis (SDM), in which a series of mutated or chimeric E^rns^ and E2 were constructed using In-Fusion HD Cloning Kit (TaKaRa, Dalian, China) according to the manufacturer’s instructions and expressed by the baculovirus system and subjected to Western blot analysis using anti-E^rns^ and anti-E2 mAbs under non-reducing conditions.

To identify the antigenic regions recognized by these mAbs, serially truncated E2 genes were amplified by PCR using the full E2 gene as the template according to the design shown in **Figure 5A**. The chimeric E2 or E^rns^ genes with substitution of the corresponding region between two CSFV strains or between CSFV and BVDV1 were also constructed by PCR according to the design shown in **Figures 5B**, **6**. The truncated and chimeric genes were baculovirus-expressed and then analyzed by Western blot as described above.

## 3 Result

### 3.1 Generation and isotype identification of mAbs against CSFV E^rns^ and E2

Using baculovirus-expressed E^rns^ and E2, five mAb hybridoma cell clones against HCLV-E^rns^ (1104, 1204, 1504, 1904, and 2004) and one against HCLV-E2 (6B211) were obtained through a series of selection and cloning processes. Using lentivirus-expressed E2, two mAb hybridoma clones against Shimen (SM) E2 (3H3G6 and 9A4H4) were obtained. Of the eight mAbs, 6B211 and 9A4H4 were published earlier (36, 47) and were included in the present study to further identify their reactivity and antigenic epitopes. The isotypes of these eight mAbs were IgG2b (3H3G6 and 9A4H4) and IgG1 (6B211, 1104, 1204, 1504, 1904, and 2004) with κ-type light chains (data not shown).

### 3.2 Specificity and capability of the anti-E2 and anti-E^rns^ mAbs to recognize and differentiate vaccine strain and field CSFVs

**Figure 1:**
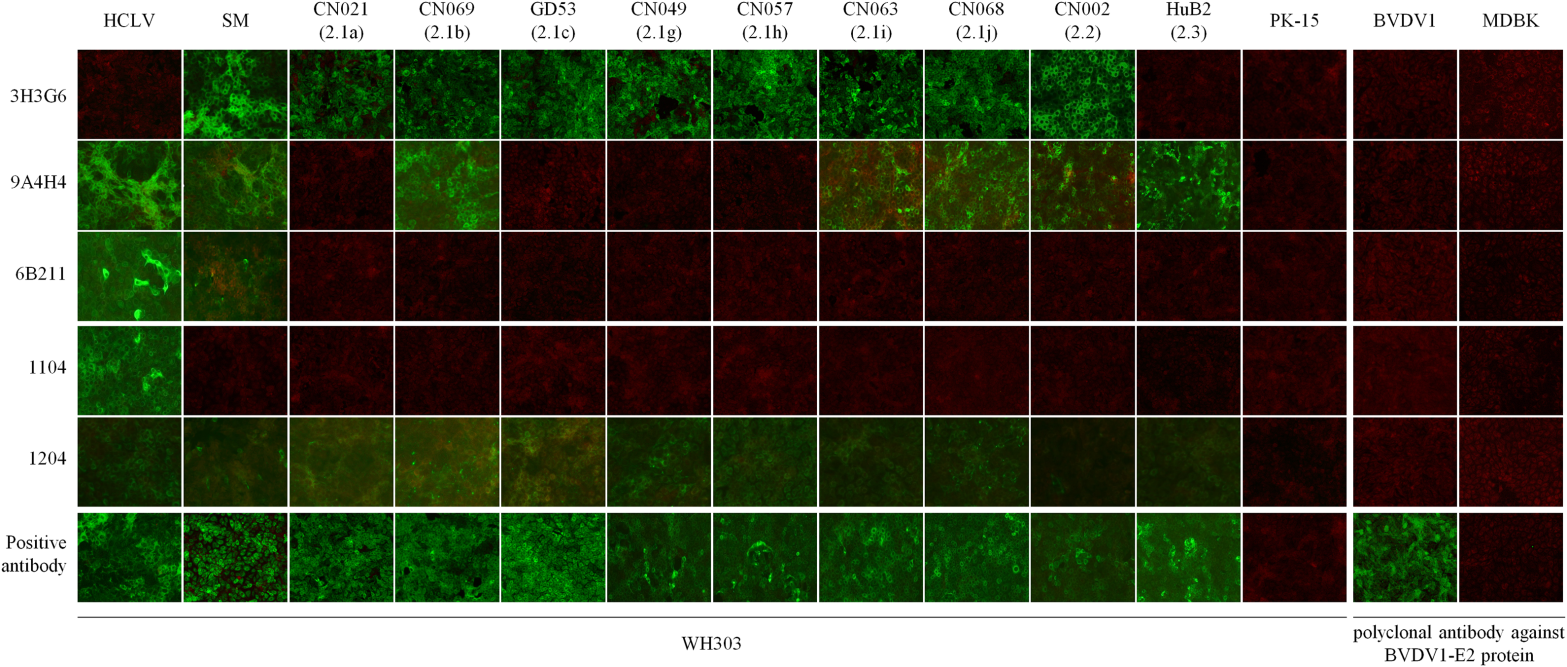
Detection of CSFVs cultured in PK-15 cells by IFA with the five mAbs. Each mAb row shows the detection of different viruses by the mAb, while each virus column shows its reaction respectively with each of the five mAbs. Bottom positive antibody row detected by CSFV universal mAb WH303 was control to confirm the growth of 11 test viruses. The PK-15 column shows negative detection of mock-infected PK-15 cells by each of the five mAbs. The right panel is used to confirm the specificity of the five mAbs, showing negative detection of BVDV1 in MDBK cells. BVDV1 growth was validated by IFA using an anti-BVDV1 E2 polyclonal antibody.

To characterize the specificity of the CSFV spectrum recognized by these mAbs, we conducted IFA on cells infected respectively with 106 CSFV field isolates belonging to different genotypes, along with reference HCLV, SM, and negative virus control BVDV. **Figure 1** shows partial IFA data, and the overall result is summarized in **Table 1** and **Table S2**. All eight mAbs could recognize different CSFV strains, but not BVDV1, indicating that they are all CSFV-specific. The anti-E^rns^ mAb 1104, 1504, 1904, and 2004 reacted only with the HCLV vaccine strain, not with the virulent SM strain or any other field isolates, indicating that they were highly vaccine-specific and capable to differentiate HCLV from SM and field isolates.

**Table 1:** Reactivity of HCLV, SM, and 106 field isolates with eight mAbs in IFA.

	Strain and genotype	Anti-E2 mAb			Anti-E^rns^ mAb				
		3H3G6	9A4H4	6B211	1104	1504	1904	2004	1204
**Reference strains**	SM(1.1)	1/1	1/1	1/1	0/1	0/1	0/1	0/1	1/1
	HCLV(1.1)	0/1	1/1	1/1	1/1	1/1	1/1	1/1	1/1
**CSFV field isolates**	1.1	2/2	2/2	2/2	0/2	0/2	0/2	0/2	2/2
	2.1a	8/8	0/8	0/8	0/8	0/8	0/8	0/8	8/8
	2.1b	14/15	15/15	0/15	0/15	0/15	0/15	0/15	15/15
	2.1c	22/22	0/22	0/22	0/22	0/22	0/22	0/22	22/22
	2.1g	2/2	0/2	0/2	0/2	0/2	0/2	0/2	2/2
	2.1h	11/17	1/17	0/17	0/17	0/17	0/17	0/17	17/17
	2.1i	3/3	3/3	0/3	0/3	0/3	0/3	0/3	3/3
	2.1j	1/1	1/1	0/1	0/1	0/1	0/1	0/1	1/1
	2.2	17/24	23/24	8/24	0/24	0/24	0/24	0/24	24/24
	2.3	0/12	12/12	0/12	0/12	0/12	0/12	0/12	12/12
	**Total**	81/108	59/108	12/108	1/108	1/108	1/108	1/108	108/108

In contrast, anti-E^rns^ mAb 1204 reacted with HCLV and SM strains, as well as all field isolates. Furthermore, the anti-E2 mAb 3H3G6 reacted specifically with the SM strain and most field isolates (80/106) within genotypes 1 and 2, but not with the HCLV strain, indicating that it was field isolate-specific with the capability to differentiate most field isolates from HCLV. The anti-E2 mAb 9A4H4 reacted broadly with SM, HCLV, and about half of the field isolates (57/106), while anti-E2 mAb 6B211 reacted with SM, HCLV, and a limited number of field isolates (10/106) within sub-genotypes 1.1 and 2.2.

**Figure 2:**
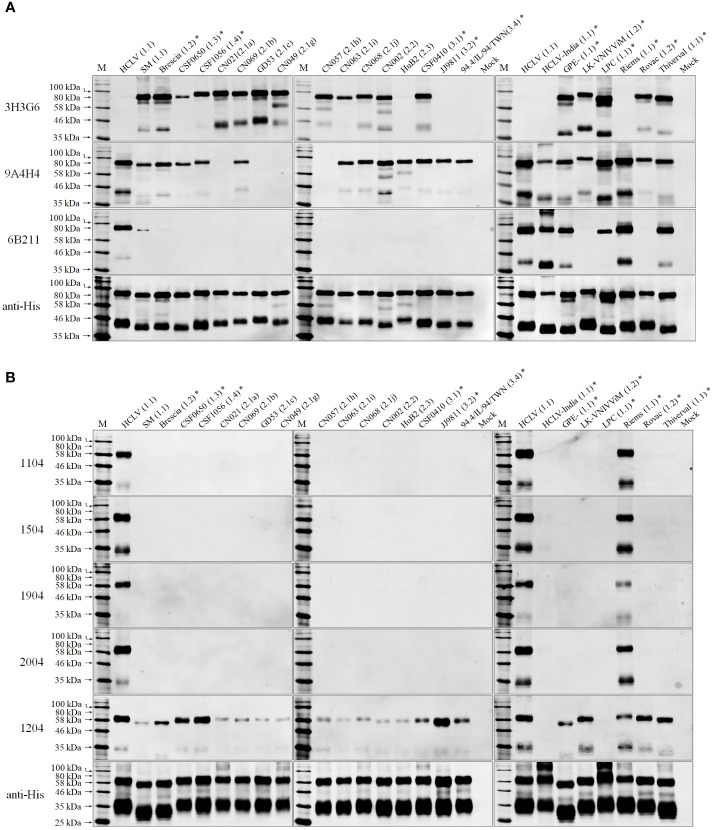
Western blot analysis of baculovirus-expressed E2 and E^rns^ of 24 selected CSFVs representing various sub-genotypes in **Figure S1**: **(A)**, the reactivity of E2 with three anti-E2 mAbs; **(B)**, the reactivity of E^rns^ with five anti-E^rns^ mAbs. The result of 11 CSFVs available in our laboratory is completely consistent with that in **Figure 1**. The bottom panel is the normalization of two proteins loaded for each lane as detected by anti-His mAb. *: the viruses were not available in our laboratory.

To confirm the above result and further characterize the differentiating capability of the mAbs, we selected 24 CSFVs representing 10 sub-genotypes in the world except for sub-genotype 3.3 (its full E2 and E^rns^ gene sequences are not available online), including different vaccine strains (**Figure S1**), and produced corresponding E^rns^ and E2 proteins using the baculovirus/insect cell system and evaluated the reactive spectrum of these mAbs using Western blot analysis. As shown in **Figure 2A**, the anti-SM-E2 mAb 3H3G6 reacted strongly with E2 of the SM strain and most field isolates of sub-genotypes 1.1, 1.2, 1.3, 1.4, 2.1, 2.2, and 3.1 and 5/8 vaccine strains, but not with the E2 of sub-genotype 2.3, 3.2, and 3.4 field isolates and three sub-genotype 1.1 lapinized vaccine strains (HCLV, HCLV-India, and Riems). This result showed that mAb 3H3G6 is highly field isolate-specific, able to differentiate most field isolates from lapinized vaccine strains, similar to its specificity in IFA analysis (**Figure 1**). Moreover, anti-HCLV-E2 mAb 6B211 reacted strongly with E2 of most vaccine strains, and very weakly with E2 of SM, but not with the E2 of the field isolates. Anti-SM-E2 mAb 9A4H4 had broad reactivity with most CSFV field isolates and vaccine strains (**Figure 2A**).

For HCLV-E^rns^-specific monoclonal antibodies, the mAbs 1104, 1504, 1904, and 2004 reacted only with E^rns^ of two sub-genotype 1.1 vaccine strains (HCLV and Riems) rather than field isolates of multiple genotypes and other vaccine strains (**Figure 2B**), indicating that they are more HCLV-specific. In contrast, mAb 1204 broadly reacted with E^rns^ of SM, most vaccine strains including HCLV and field isolates (**Figure 2B**).

### 3.3 Epitope typing and mapping

#### 3.3.1 Epitope typing

**Figure 3:**
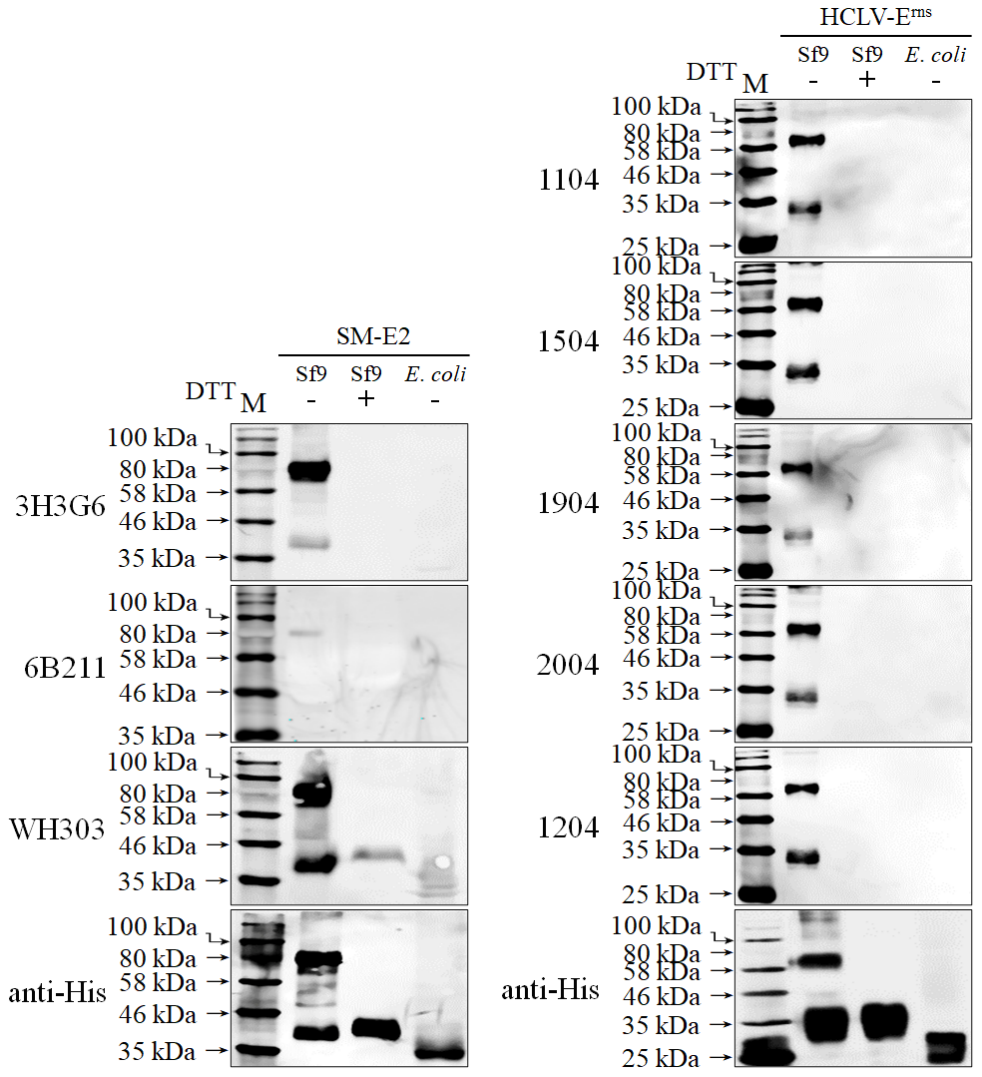
Identification of epitope type recognized by the mAbs in Western blot under reduced (DTT treatment) or non-reduced conditions. The result showed that those mAbs recognize conformational epitopes. The bottom panel is the normalization of two proteins loaded for each lane as detected by anti-His mAb.

Our previous study showed that the epitope recognized by mAb 9A4H4 was conformational (47). To identify the epitope types of the other seven mAbs, SM-E2 and HCLV-E^rns^ proteins expressed respectively with baculovirus and *E. coli* expression systems were used in Western blot. The results in **Figure 3** showed that the epitopes recognized by these seven mAbs are also conformational.

#### 3.3.2 Critical aa motifs of the epitopes recognized by anti-E2 mAbs

**Figure 4:**
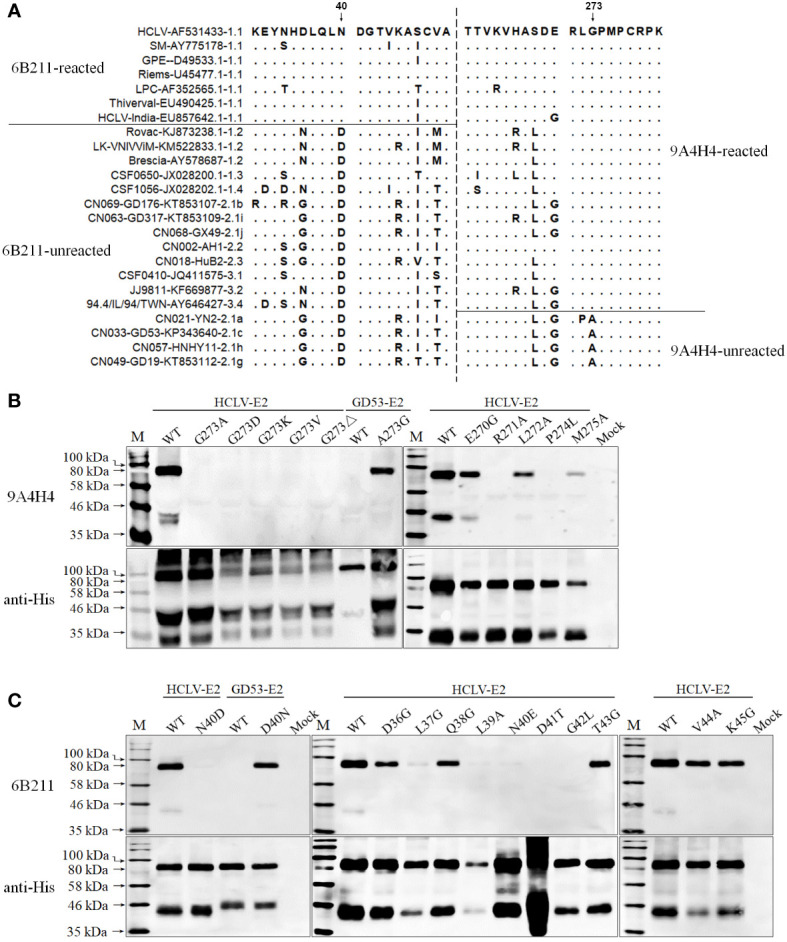
Mapping of the epitopes recognized by mAbs 9A4H4 and 6B211. **(A)**, multiple E2 sequence alignment shows consensus residues differentiating reacted and unreacted CSFVs with both mAbs in **Figure 2**. Reactivity of mAb 9A4H4 **(B)** and 6B211 **(C)** with site-directed mutated E2 proteins of vaccine HCLV and field isolate GD53. The original E2 (WT) was included as controls. The bottom panel is the normalization of mutated E2 for each lane as detected by anti-His mAb.

In order to define the critical aa motifs constituting the conformational epitopes recognized by anti-E2 mAbs, a multiple-aa-sequence alignment of E2 proteins reacted and unreacted with mAbs 9A4H4, 6B211, and 3H3G6 was conducted. The results revealed two highly uniform aa sites on E2 between reactive and non-reactive E2 proteins, the aa 273 for 9A4H4 and aa 40 for 6B211 (**Figure 4A**), but no consensus site was found for 3H3G6. The E2 that reacted with 9A4H4 has G^273^ but the unreacted one has A^273^, while the E2 that reacted with 6B211 has N^40^ but the unreacted one has D^40^ (**Figure 4A**). Therefore, E2 sequences of the reacted HCLV strain and unreacted GD53 strain were selected for site-directed mutagenesis (SDM). Western blot analysis of the mutated E2 proteins showed that mutations G^273^A, G^273^D, and G^273^K and G^273^V and G^273^ deletion (G^273^Δ) on reacted HCLV-E2 could completely abolish its reactivity with mAb 9A4H4, while A^273^G mutation of unreacted GD53-E2 could generate its reactivity with mAb 9A4H4 (**Figure 4B**). Meanwhile, mutations R^271^A and P^274^L, but not E^270^G, L^272^A, and M^275^A at flanking sites of HCLV-E2 also abolished its reactivity with 9A4H4 (**Figure 4B**). In the same way, the mutations N^40^D and N^40^E on reacted HCLV-E2 could effectively abolish or diminish its reactivity with mAb 6B211, while mutation D^40^N on unreacted GD53-E2 could generate its reactivity with mAb 6B211 (**Figure 4C**). Meanwhile, mutations L^37^G, L^39^A, D^41^T, and D^42^L, but not D^36^G, Q^38^G, T^43^G, V^44^A, and K^45^G, at flanking sites of reacted HCLV-E2 also abolished or significantly diminished its reactivity with 6B211 (**Figure 4C**). These results showed that the critical aa motifs of mAbs 9A4H4- and 6B211-recognizing epitopes are ^271^RXGP^274^ and ^37^LXLNDG^42^, respectively.

**Figure 5:**
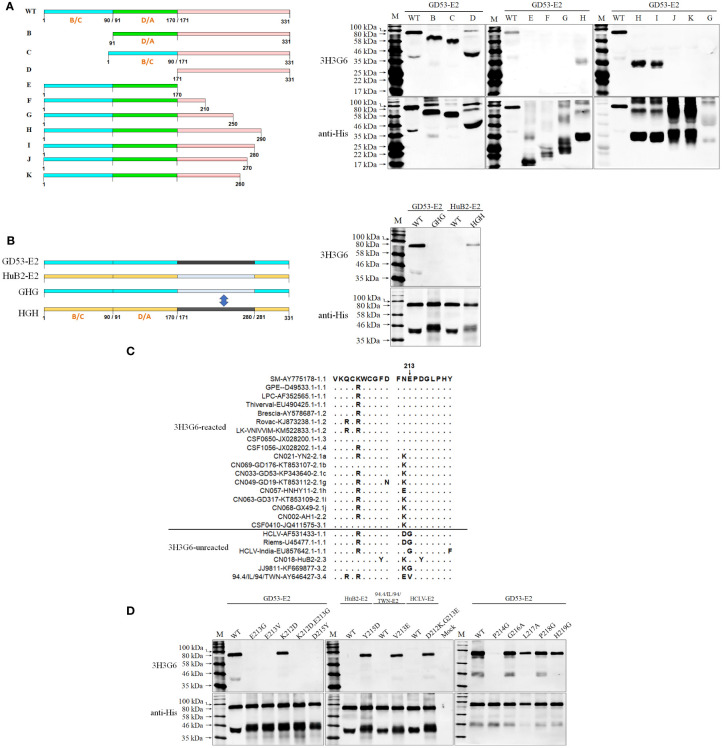
Mapping of the epitope recognized by mAb 3H3G6. **(A)**, determination of the E2 region reacted with 3H3G6 by Western blot analysis of 10 truncated E2 fragments (B–K). **(B)**, confirmation of E2 regions 171–280 aa by Western blot of chimeric E2 proteins with the region swapped between reacted GD53 and unreacted HuB2 isolates. **(C)**, multiple E2 sequence alignment of 3H3G6-reacted and -unreacted CSFVs in **Figure 2** shows candidate sites for SDM. **(D)**, reactivity of 3H3G6 with site-directed mutated E2 proteins of vaccine HCLV and field isolates GD53, HuB2, and 94.4/IL/94/TWN. The original E2 (WT) was included as controls. The bottom panel is the normalization of mutated E2 for each lane as detected by anti-His mAb.

Since the multiple-aa-sequence alignment of E2 proteins reacted and unreacted with mAb 3H3G6 did not yield a consensus site for SDM, three fragments (B-D) of the GD53-E2 protein with deletions of different antigenic regions (13, 48–50) were expressed in the baculovirus system and subjected to Western blot analysis (**Figure 5A**). The result showed that 3H3G6 reacted with all three fragments E2-ΔB/C (aa 1–90), E2-ΔD/A (aa 91–170), and E2-ΔB/C-ΔD/A (aa 1–170), indicating that its recognizing epitope is located at the C-terminal half of E2. In further mapping, serial GD53-E2 fragments truncated from the C terminal (E-K) were constructed for Western blot analysis, and the result showed the reactivity of 3H3G6 with only fragments H (aa 1–290) and I (aa 1–280). These results suggested that the 3H3G6-recognizing epitope was located in the 171–280-aa region of the E2 protein.

Furthermore, a swap of this region (aa 171–280) between reacted GD53-E2 and unreacted HuB2-E2 was conducted for Western blot and the result showed that the swapping successfully reversed the reactivity with 3H3G6 between GD53-E2 and HuB2-E2 (**Figure 5B**), eventually confirming that the location of the 3H3G6-recognizing epitope is in the 171–280-aa region. To finally define the critical aa motif constituting the epitope, the multiple-aa-sequence alignment of the 171–280-aa E2 regions between the reacted and unreacted helped us identify several candidate aa sites for follow-up SDM (**Figure 5C**). The result in **Figure 5D** showed that mutations E^213^G, E^213^V, KE^213/214^EG, and D^215^Y on reacted GD53-E2 abolished its reactivity with 3H3G6, while mutations Y^215^D, V^213^E, and DG^212/213^KE respectively on unreacted HuB2-E2, 94.4/IL/94/TWN-E2, and HCLV-E2 brought them the reactivity. In addition, mutation at conservative site 214 (P to G) but not sites 216 to 219 also abolished the reactivity of GD53-E2 with 3H3G6. These results revealed that the critical aa motif of the 3H3G6 recognizing epitope is ^213^EPD^215^ on E2.

#### 3.3.3 Critical aa motifs of epitopes recognized by anti-E^rns^ mAbs

**Figure 6:**
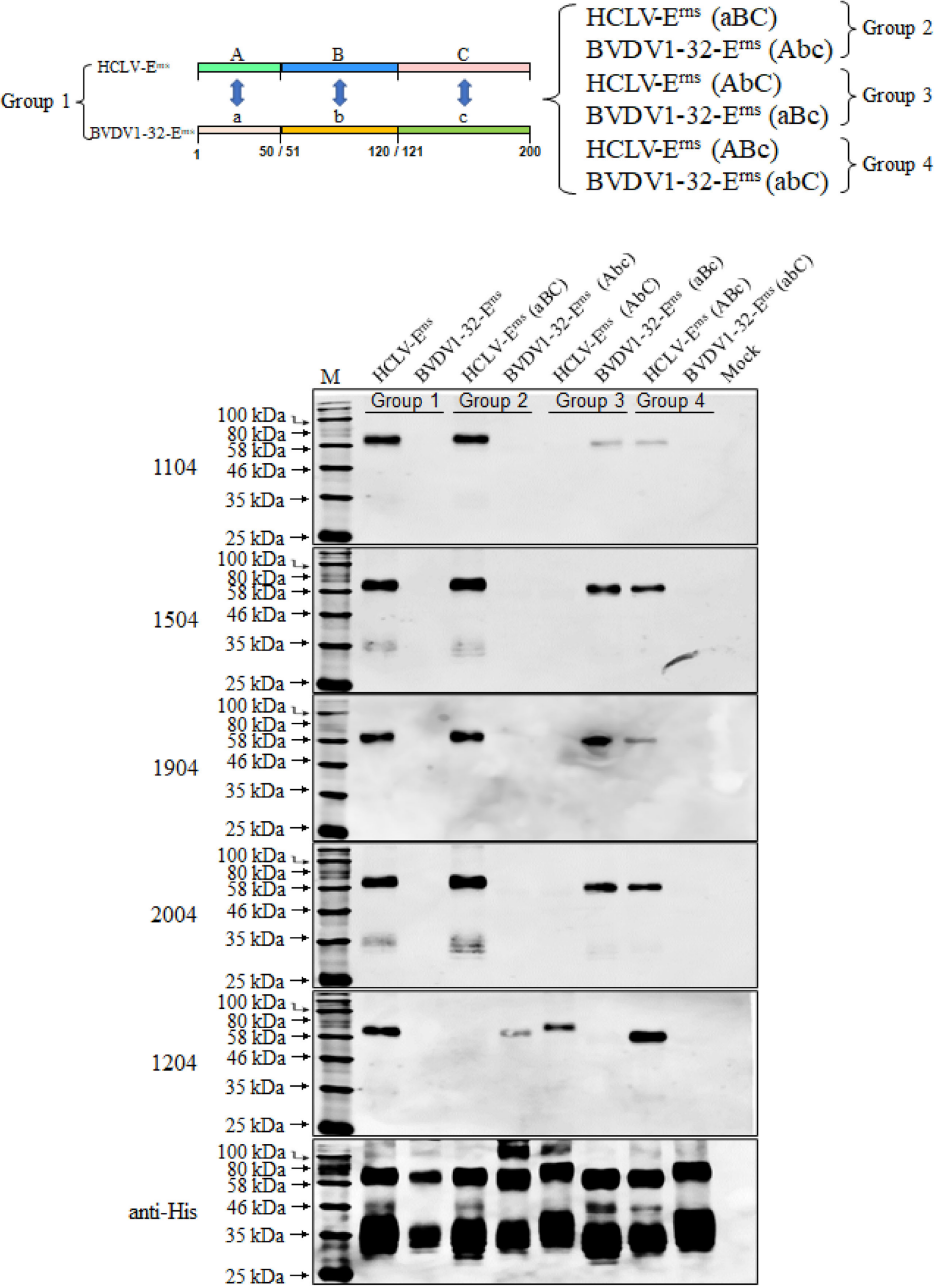
Determination of the E^rns^ region recognized by five anti-E^rns^ mAbs. E^rns^ chimera with A (aa 1–50), B (aa 51–120), and C (aa 121–200) regions respectively swapped between reacted HCLV-E^rns^ and unreacted BVDV1-32-E^rns^ were constructed to identify the reactive region in Western blot. It showed that the epitope recognized by 1204 is located in region A (aa 1–50), while that recognized by 1104, 1504, 1904, and 2004 is located in region B (aa 51–120). The original E^rns^ proteins of HCLV and BVDV1-32 were included as controls. The bottom panel is the normalization of E^rns^ chimera for each lane as detected by anti-His mAb.

To identify the epitopes and critical aa sites recognized by E^rns^ mAbs, the full-length E^rns^ proteins (200-aa except C-terminal anchor peptide) of all strains in **Figure 2B** were aligned, but no consensus aa sites were found between the reacted and unreacted strains (**Figure S2**). Thus, serial chimeric E^rns^ proteins with a mutual substitution of corresponding regions between HCLV and BVDV were constructed and expressed in the baculovirus system for Western blot analysis. As depicted in **Figure 6**, mAb 1204 reacted with chimeric BVDV1-32-E^rns^ (Abc), but not with HCLV-E^rns^ (aBC) in group 2, and mAbs 1104, 1504, 1904, and 2004 reacted with BVDV1-32-E^rns^ (aBc), but not with HCLV-E^rns^ (AbC) in group 3. These results showed that the mAb 1204-recognizing epitope was located in the A region (aa 1–50) of HCLV-E^rns^, while that of four other mAbs was located in B region (aa 51–120) of HCLV-E^rns^ (**Figure S2**).

**Figure 7:**
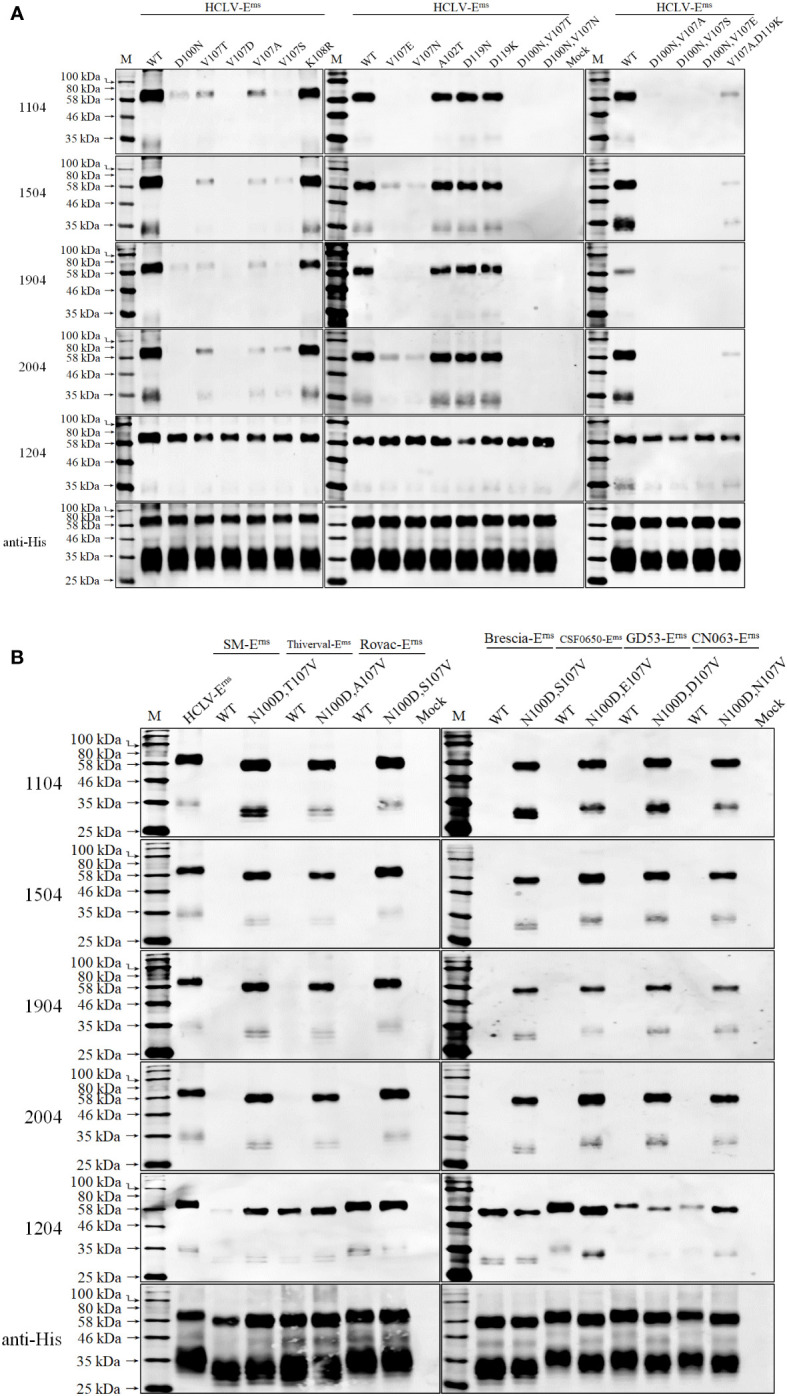
Epitope mapping and validation. **(A)**, the critical amino acids of the epitopes recognized by five anti-E^rns^ mAbs were defined on HCLV-E^rns^. SDM results showed that single mutations of D^100^ or V^107^ of HCLV-E^rns^ correspondingly to the residues of field isolates diminish or abolish the reactivity with four mAbs rather than 1204, while double mutations at the two sites could completely abolish the reactivity. **(B)**, the original residues 100 and 107 of seven unreacted isolate E^rns^ proteins were changed to N^100^ and V^107^ of HCLV-E^rns^ whose reactivity was conferred with four mAbs rather than 1204. All mutations had no influence on mAb 1204. The original E^rns^ proteins of HCLV and seven field isolates (WT) were included as controls. The bottom panel is the normalization of the E^rns^ chimera for each lane as detected by anti-His mAb.

The above observation revealed a short region for SDM to define the key aa motifs. Based on B-region (aa 51–120) sequence alignment, aa at sites 100, 102, 107, and 119 of HCLV-E^rns^ were respectively replaced with corresponding aa of 1104-unreacted strains and the Western blot in **Figure 7A** showed that single mutations at D^100^ and V^107^ of HCLV-E^rns^ significantly diminished E^rns^ reactivity with mAbs 1104, 1504, 1904, and 2004, while double mutations at the two sites completely abolished the reactivity (D^100^N/V^107^T, D^100^N/V^107^N, D^100^N/V^107^T, D^100^N/V^107^N, D^100^N/V^107^S, and D^100^N/V^107^E). However, the mutations at sites 102 and 119 had no impact. All these mutations had no impact on mAb 1204. To confirm the result, simultaneous mutations of these two sites from the aa of six unreacted strains to D^100^/V^107^ of HCLV successfully rendered E^rns^ of six unreacted strains the same strong reactivity (**Figure 7B**). These results undoubtedly showed that D^100^ and V^107^ are critical amino acids of the epitope recognized by mAbs 1104, 1504, 1904, and 2004.

It is interesting to note that both 1104 group and 1204 did not react with E^rns^ of the HCLV-Indian strain although it has the same D^100^ and V^107^. HCLV-Indian E^rns^ has a minimum aa difference with Chinese HCLV (only a five-aa difference at sites 44, 49, 53, 87, and 92 in A (aa 1–50) and B (aa 51–120) regions as shown in **Figure S2**). To delineate if these five different amino acids abolished the reactivity, their reciprocal substitution between HCLV-India and Chinese HCLV was done separately, and the result showed that the reactivity of HCLV-E^rns^ with mAbs 1104 group and 1204 was not altered as the aa residue 44, 49, 53, or 87 was mutated to that of HCLV-Indian E^rns,^ but was significantly diminished with the mAb 1104 group or abolished with mAb 1204 as its aa residue 92 mutated to that of HCLV-Indian E^rns^ (i.e., Q^92^P mutation). In contrast, the reactivity of HCLV-Indian E^rns^ with the mAbs 1104 group and 1204 was successfully rescued as its aa residue 87 or 92 was mutated to that of HCLV-E^rns^ (i.e., V^87^I or P^92^Q), rather than mutations of residues 44, 49, and 53 to those of HCLV-E^rns^ (**Figure S3**).

**Figure 8:**
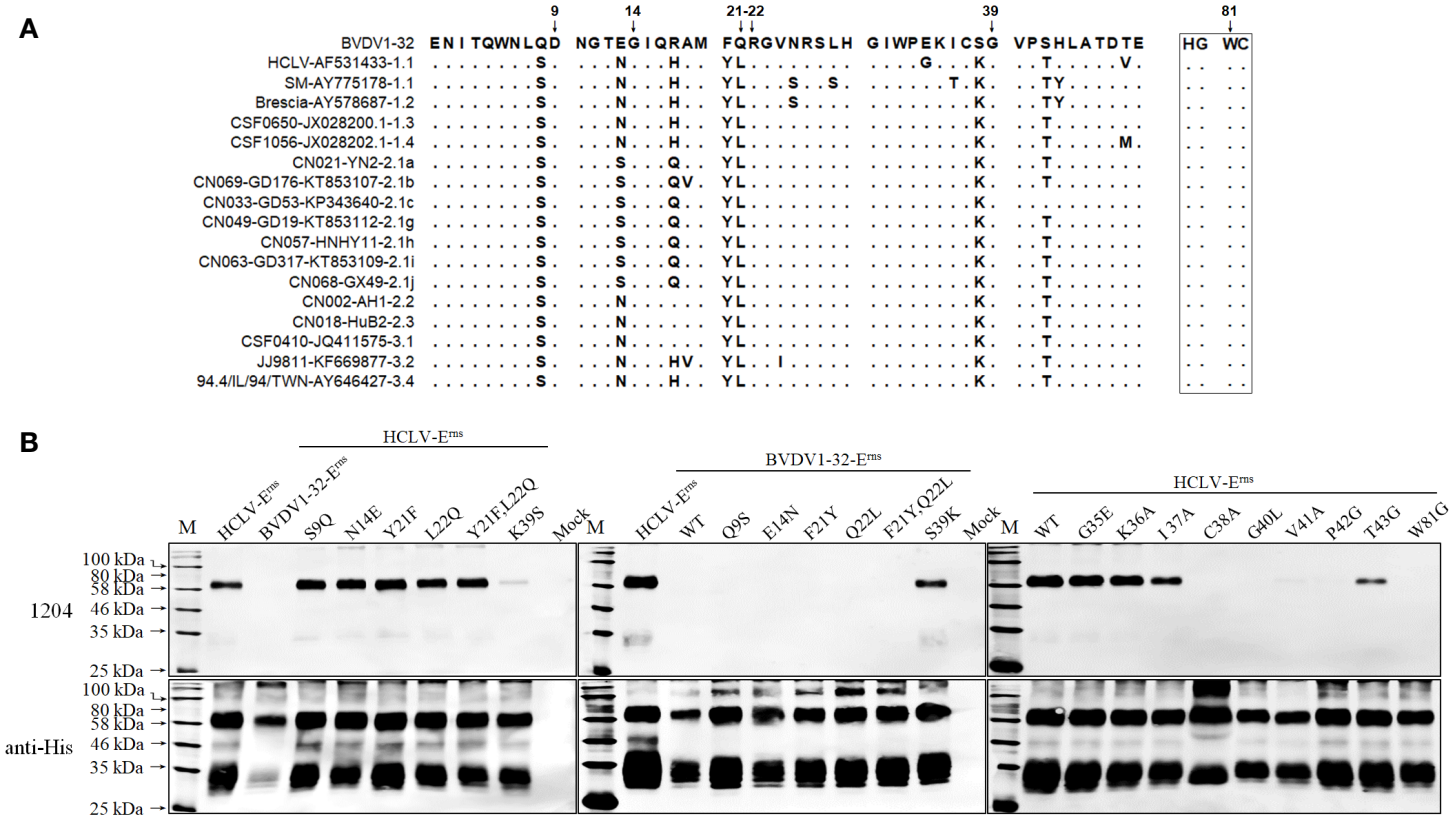
Mapping of the epitope recognized by mAb 1204. **(A)**, multiple E^rns^ sequence alignment of 1204-reacted CSFVs and -unreacted BVDV1-32 shows candidate sites for SDM. **(B)**, reactivity of 1204 with site-directed mutated E^rns^ proteins respectively of HCLV and BVDV1-32. The native E^rns^ proteins (WT) of both viruses were included as controls. The bottom panel is the normalization of E^rns^ proteins for each lane as detected by anti-His mAb.

**Figure 9:**
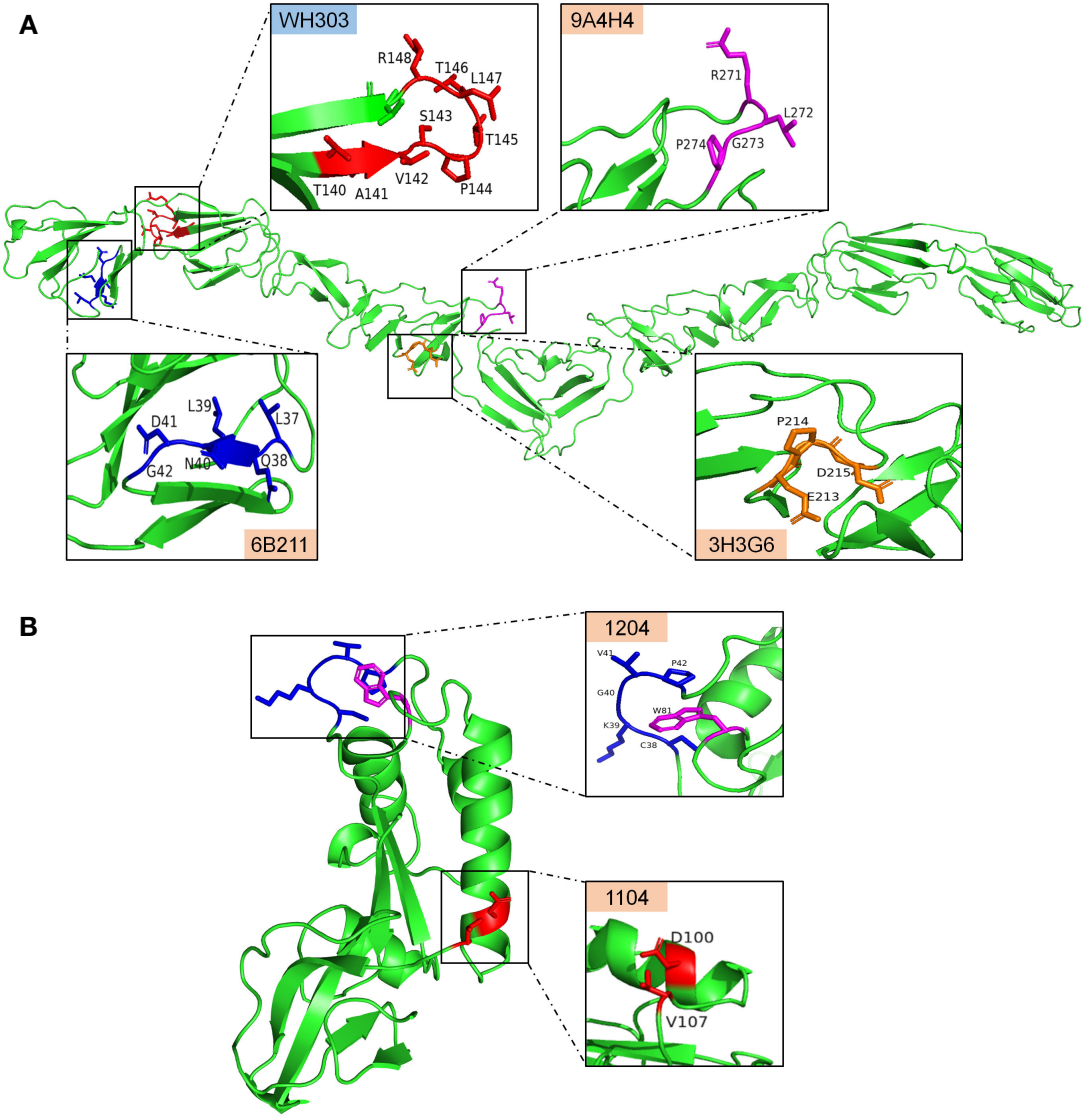
The antigenic epitopes recognized by mAbs identified in this study and WH303 on the predicted crystal structure of CSFV proteins created with PyMOL. **(A)**, antigenic epitopes recognized by 3H3G6, 6B211, and 9A4H4 were mapped on the E2 protein structure of the CSFV Shimen strain, and the known CSFV conservative epitope recognized by WH303 was set as a reference. **(B)**, antigenic epitopes recognized by mAbs 1104 and 1204 were mapped on the structure of HCLV-E^rns^ protein.

To further define the epitope motif recognized by mAb 1204, the reciprocal substitution of distinct aa residues in the A region (aa 1–50) between reacted HCLV and unreacted BVDV-E^rns^ proteins was done separately for Western blot analysis, and the result in **Figure 8** showed that the reactivity of HCLV-E^rns^ with mAb 1204 was significantly diminished only with the K^39^S mutation, while the reactivity of BVDV-E^rns^ with the mAb was successfully rescued only with the S^39^K mutation, indicating that K^39^ is critical in the epitope recognized by mAb 1204. More mutations at flanking sites of K^39^ further showed that aa residues C^38^, G^40^, V^41^, P^42^, and W^81^, which are structurally close to K^39^ (**Figure 9B**), are also important residues constituting the epitope. These results indicate that aa sequence ^38^CKGVP^42^,W^81^ completely conservative among CSFVs is the critical motif of the E^rns^ epitope recognized by mAb 1204 (**Figure S2**).

## 4 Discussion

The antigenic structure of CSFV E2 has been extensively studied, and some epitopes or critical aa motifs, both linear and conformational including neutralizing ones, such as the CSFV-specific mAb WH303 recognizing the conservative linear epitope ^140^TAVSPTTLR^148^ (**Figure 9A**), have been identified with the most of them located in N and a few in C terminal halves (9, 34). An important finding in the present study is the identification of three novel conformational E2 epitopes by using three newly generated anti-E2 mAbs. The anti-SM-E2 mAb 3H3G6 reacted with the most field isolates but not HCLV or the other three lapinized vaccine E2 proteins (HCLV, HCLV-India, and Riems). Since 2000, the sub-genotype 2.1, particularly sub-sub-genotypes 2.1b and 2.1c, has become dominant in Mainland China, while sub-genotypes 2.2 and 2.3 have become silent (51). In this case, the dominant field strain-specific mAb 3H3G6 enables the differentiation of field isolates from the HCLV vaccine widely used in China.

Further epitope mapping showed that 3H3G6 recognized a conformational epitope located in the C-terminal half of E2, which contains the critical motif ^213^EPD^21^, and mutation at any site of this motif could abrogate its reactivity with 3H3G6. The E2 aa sequence alignment of 106 reacted and unreacted field isolates in IFA validated the motif, showing that the 81 3H3G6-reacted isolates had ^213^EPD^215^, while the 26 3H3G6-unreacted isolates had ^213^EPY^215^ (n = 13), ^213^GPD^215^ (n = 9), or ^213^DPD^215^ (n = 5) (**Table S2**). Chang et al. reported that ^213^E is a critical determinant for the anti-E2 mAb T11, generated from a virulent sub-genotype 2.1 isolate, to recognize field isolates, while the fine epitope mapping and comprehensive viral spectrum of this mAb were not analyzed and its capacity of differentiating vaccine from field strains was not reported (50).

Unlike 3H3G6 with the field isolate specificity, anti-HCLV-E2 mAb 6B211 showed a high-level specificity for CSF vaccine strains, which reacted strongly with six vaccine strains of sub-genotype 1.1, but not with two vaccine strains of sub-genotype 1.2 (LK-VNIVViM and Rovac). It did not react with most field isolates except a minor part of sub-genotype 1.1 and 2.2 isolates and weakly with the SM strain, indicating that 6B211 enables differentiation of vaccine strains from most field isolates. In contrast, the anti-SM-E2 mAb 9A4H4 recognizes a broad spectrum of CSFVs. It reacted with the reference SM strain, most field isolates (including genotype 3), and all vaccine strains, except for a minor part of sub-genotype 2.1a, 2.1c, 2.1g, and 2.1h isolates.

The above antigenic properties showed that 3H3G6 is field isolate-specific, 6B211 is vaccine-specific, and 9A4H4 is broadly CSFV-specific. Therefore, these three mAbs constitute the most ideal mAb panel to identify the antigenic differences among CSFVs, particularly to identify the antigenic motifs specific for field isolates and vaccine strains. For this purpose, the E2 aa sequences of reacted and unreacted CSFVs in Western blot were aligned separately for each mAb, which did not find significantly consensus aa residues between 3H3G6-reacted and -unreacted viruses, but those for 9A4H4 and 6B211 revealed two highly consensus aa residues, G^273^ for 9A4H4-reacted and N^40^ for 6B211-reacted viruses, while A^273^ and D^40^ correspondingly for unreacted viruses in Western blot analysis (**Figure 4**). The characterization of epitopes and the aa sequence alignment of CSFV isolates in IFA confirmed the result (**Table 1** and **Table S2**), showing that 57 9A4H4-reacted isolates had residue G^273^, while the remaining 49 9A4H4-unreacted had A^273^. All 10 6B211-reacted isolates had N^40^, while the remaining 96 6B211-unreacted had D^40^ (n = 91) or E^40^ (n = 5) (**Table S2**). It is interesting to note a previous study reporting that D^40^ was critical for antigenic specificity of field isolate E2, and its mutation to N^40^ of vaccine strain E2 abolished its reactivity with the field isolate-derived mAb T23 (49). To define the critical aa residues for binding the mAbs, an SDM on positions 40 and 273 and their flanking residues was conducted, resulting in the identification of ^271^RXGP^274^ and ^37^LXLNDG^42^ as specific motifs recognized by 9A4H4 and 6B211, respectively (**Figure 9A**). Based on the results, residues G^273^ and N^40^ have been therefore confirmed as the antigen-specific markers, respectively, of field isolates and vaccine strains.

The antigenic structure of E^rns^ has not been studied as extensively as that of E2; at present, only eight epitopes in E^rns^ have been mapped by mAbs—they are ^31^GIWPEKIC^38^, ^65^NYTCCKLQ^72^, ^73^RHEWNKHGW^81^, ^88^DPWIQLMNR^96^, ^116^YDKNTDVNV^124^, ^127^QARNRPTT^134^, ^145^SFAGTVIE^152^, and ^161^VEDILY^166^ (23–25, 52). The present study has identified two conformational epitopes. The epitope recognized by mAb 1204 is universal for CSFVs since its critical motif ^38^CKGVP^42^,W^81^ is completely conservative among different sub-genotypes (**Figure 9B**, **Figure S2**, and **Table S2**). This motif is likely overlapped by the published linear epitope ^31^GIWPEKIC^38^, indicating that the aa 31–42 is an important antigenic region. The epitope recognized by the mAb 1104 group is highly HCLV vaccine-specific with the aa combination of D^100^/V^107^ as a critical determinant (**Figures 7**, **9B**); therefore, the mAb it elicits is able to differentiate vaccine strains from field isolates. It is interesting to note that residue 107 is also critical for antibody binding. Meyer et al. reported that A^107^ is critical for a wild-type Alfort 187 E^rns^ and mutations A^107^ to D^107^ could abolish its reaction with an Alfort 187-derived anti-E^rns^ mAb (52). The E^rns^ aa sequence alignment of various sub-genotypes showed that residue 107 is highly variable (**Figure S2**). The E^rns^ aa sequences of 108 CSFVs in **Table 1** showed that 107 1104-unreacted viruses had a quite different combination of residues 100 and 107; they are N^100^\D^107^ (n = 90), N^100^\E^107^ (n = 4), N^100^\G^107^ (n = 2), N^100^\N^107^ (n = 4), N^100^\T^107^ (n = 1), N^100^\V^107^ (n = 5), and S^100^\N^107^ (n = 1) (**Table S2**), suggesting that the antigenicity of E^rns^ alters readily upon aa mutation at residue 107.

Host immune response is an important factor to drive viral evolution through point mutations and positive selections, which usually results in emerging of viral escape mutants (9, 53). Attenuated live vaccines have been used widely since the 1950s; CSFV mutants evading immune response have been reported in recent years. These escape mutants were deemed to evolve from positive selection pressure mainly acting on CSFV E2 and E^rns^ genes, and some positively selected aa sites on the antigenic region of the structural glycoprotein have been identified by sequence-based site-by-site analysis of the dN/dS ratio (54–56). However, to confirm whether these software-identified sites were indeed positively selected under immune pressure is very difficult. The present study and a previous publication have likely identified a positively selected antigenic aa site representing CSFV evasion of humoral immune response. The vaccine E2-derived mAb 6B211 in the present study recognized only vaccine E2 with N^40^ rather than field isolate E2 with D^40^, while Chang et al. (2010) reported that T23, an anti-E2 mAb against a 1994 field isolate, recognized only field isolate E2 with D^40^ rather than vaccine E2 with N^40^ (49). The alignment of E2 sequences from GenBank showed that recent field isolates sequenced since the 1990s have overwhelming D^40^, rarely N^40^ (data not shown). In addition, 6B211-reacted isolates in **Table 1** were genotypes 1.1 and 2.2 from the 1990s, which had N^40^, but their prevalence has been largely decreased (genotype 1.1) and ceased (genotype 2.2) since 2000, while all 6B211-unreacted isolates dominating the CSF endemic in China had D^40^. Additional *in vitro* neutralization test showed that 6B211 could neutralize the CSFV isolates reacted with it but not neutralize the isolates unreacted with it in the Western blot (data not shown). These results revealed the first aa site evolving from immune evasion, which is D^40^ in E2 and can be considered as the positively selected marker residue of CSFV field isolates under immune pressure.

In summary, we have generated and systematically characterized field isolate-specific, vaccine strain-specific, and all CSFV-specific mAbs. Our studies have revealed five novel conformational antigenic epitopes on E2 and E^rns^. The critical aa motifs of these epitopes are residue ^213^E of E2 in the field isolate, and residues N^40^ of E2 and D^100^/V^107^ of E^rns^ are in vaccine strains. N^40^D mutation on E2 might occur naturally under positive selection, resulting in CSFV evasion of host immune response. These findings provide new insights into the antigenic structure and antigenic evolution of CSFV E2 and E^rns^ glycoproteins. mAbs generated in our study are valuable tools for the development of novel diagnostic assays, particularly for the differentiation between field isolates and vaccine strains, or between vaccinated and naturally infected animals in the eradication process of CSF.

## Data availability statement

The original contributions presented in the study are included in the article/**Supplementary Material**. Further inquiries can be directed to the corresponding authors.

## Ethics statement

The animal study was reviewed and approved by Institutional Animal Care and Use Committee at Kansas State University.

## Author contributions

SM, LW, HL, FB, LZ, WG, JS, and CT contributed to conception and design of the study. SM, FB, and WG contributed to the isolation and identification of CSFV filed strains. LW, HL, RM, YM, and RY contributed to the preparation of monoclonal antibodies. LW, HL, XS, XX, WG, JS, and CT organized the database. SM, WG, and CT wrote the draft of the manuscript. All authors contributed to manuscript revision, read, and approved the submitted version.

## Funding

This work was supported by the following grants: National Natural Science Foundation of China to WG (32072843) and the National Key Research and Development Program of China to CT (2017YFD0500103) and to XS (2021YFF0703800). This work is also supported by awards from the National Bio and Agro-Defense Facility Transition Fund, the USDA National Institute of Food and Agriculture, Hatch-Multistate project, grant number [1021491], and National Pork Board Grant, grant number [18-059].

## Conflict of interest

Author RY is employed by Guangzhou Bioneeds Biotechnology Co., Ltd., China.

The remaining authors declare that the research was conducted in the absence of any commercial or financial relationships that could be construed as a potential conflict of interest.

## Publisher’s note

All claims expressed in this article are solely those of the authors and do not necessarily represent those of their affiliated organizations, or those of the publisher, the editors and the reviewers. Any product that may be evaluated in this article, or claim that may be made by its manufacturer, is not guaranteed or endorsed by the publisher.

## Supplementary material

Supplementary Figure 1 Full E2 gene-based phylogenetic analysis of representative CSFVs of all 11 subgenotypes. Twenty four reference strains and field isolates used in western blot in **Figure 2** were Icon-labeled, which represent different genetic branches of 10 sub-genotypes except for sub-genotype 3.3 (its full E2 not available). ▲: available field isolates; ◆: available reference virulent SM and HCLV vaccine strains; ■: unavailable field isolates; ●: unavailable vaccine strains.

Supplementary Figure 2 Alignment of full E^rns^ sequence (200 aa without C-terminal anchor peptide) of all reacted and unreacted CSFVs with 5 anti-E^rns^ mAbs in **Figure 2B**. The mAb 1204 reacted with all CSFVs, but HCLV-India and LPC vaccine strains, while 1104 other 3 mAbs only reacted with tope two vaccine strains HCLV and Riems. The sequence of BVDV1-32 is included for comparison in order to construct E^rns^ chimera based on mutual substitution of randomly defined **(A–C)** fragments between HCLV and BVDV in **Figure 6**.

Supplementary Figure 3 Determination of the critical residues responsible for difference of E^rns^ reactivity with 5 anti-E^rns^ mAbs between Chinese HCLV and HCLV-India. Bottom panel is the normalization of mutated E^rns^ proteins for each lane as detected by anti-His mAb.
