# Supplementary figures and images for: Characterization of monoclonal antibodies that specifically differentiate field isolates from vaccine strains of classical swine fever virus
Source: Front Immunol. 2022 Jul 19;13:930631. doi: 10.3389/fimmu.2022.930631 (PMC9361847; doi:10.3389/fimmu.2022.930631)

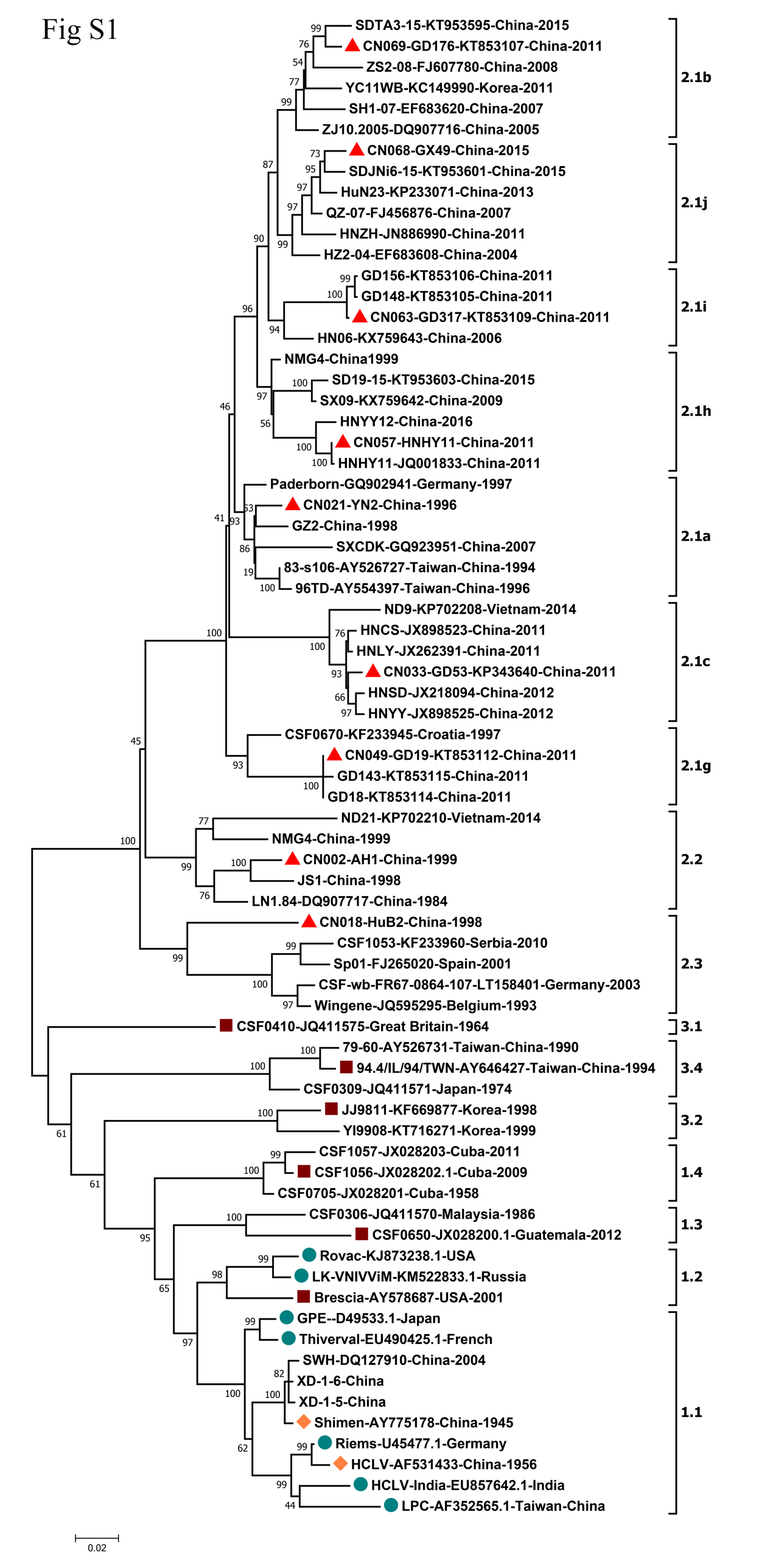

Supplement: Supplementary Figure 1 — Full E2 gene-based phylogenetic analysis of representative CSFVs of all 11 subgenotypes. Twenty four reference strains and field isolates used in western blot in Figure 2 were Icon-labeled, which represent different genetic branches of 10 sub-genotypes except for sub-genotype 3.3 (its full E2 not available). ▲: available field isolates; ◆: available reference virulent SM and HCLV vaccine strains; ■: unavailable field isolates; ●: unavailable vaccine strains. [file Image_1.tif]

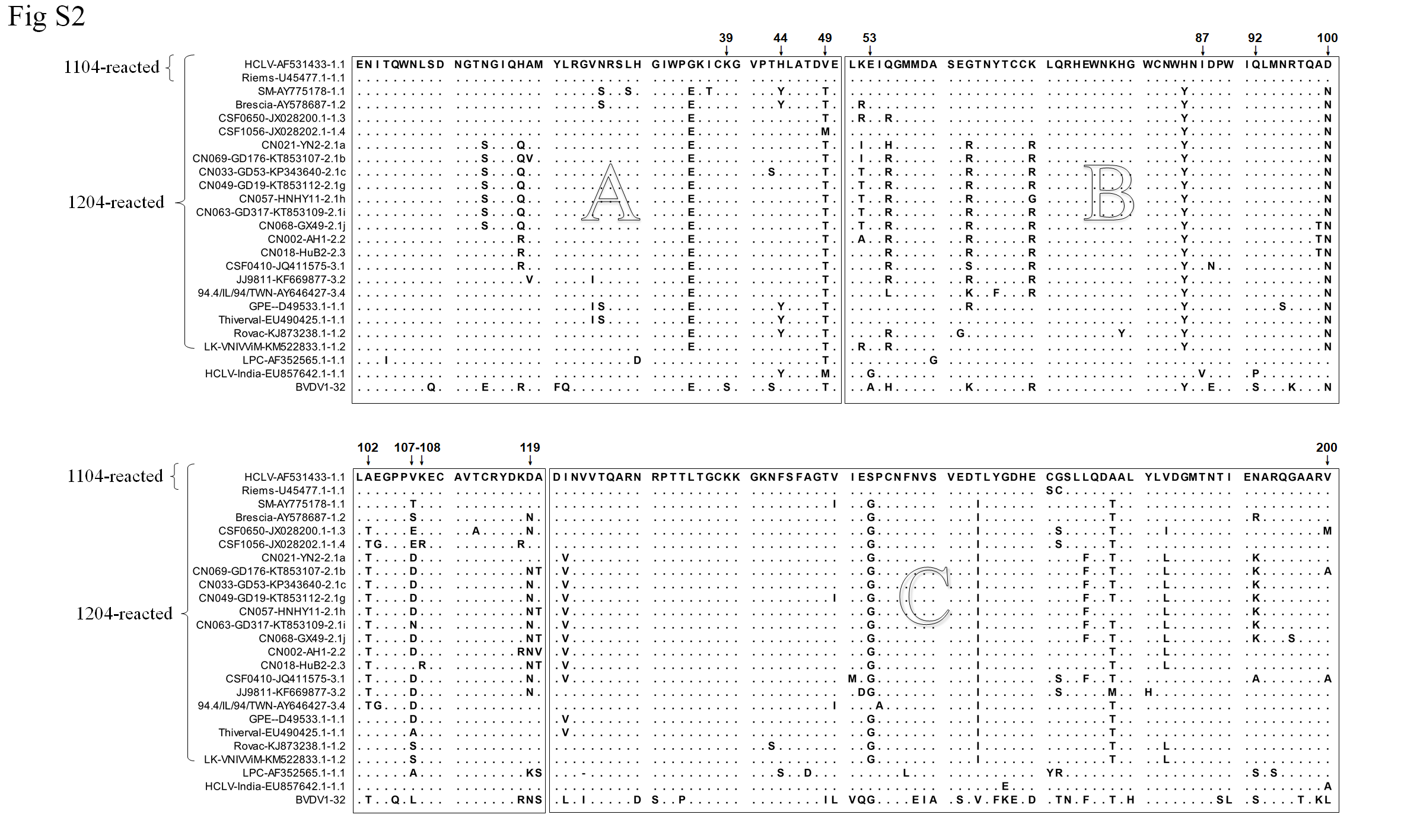

Supplement: Supplementary Figure 2 — Alignment of full Erns sequence (200 aa without C-terminal anchor peptide) of all reacted and unreacted CSFVs with 5 anti-Erns mAbs in Figure 2B . The mAb 1204 reacted with all CSFVs, but HCLV-India and LPC vaccine strains, while 1104 other 3 mAbs only reacted with tope two vaccine strains HCLV and Riems. The sequence of BVDV1-32 is included for comparison in order to construct Erns chimera based on mutual substitution of randomly defined (A–C) fragments between HCLV and BVDV in Figure 6 . [file Image_2.tif]

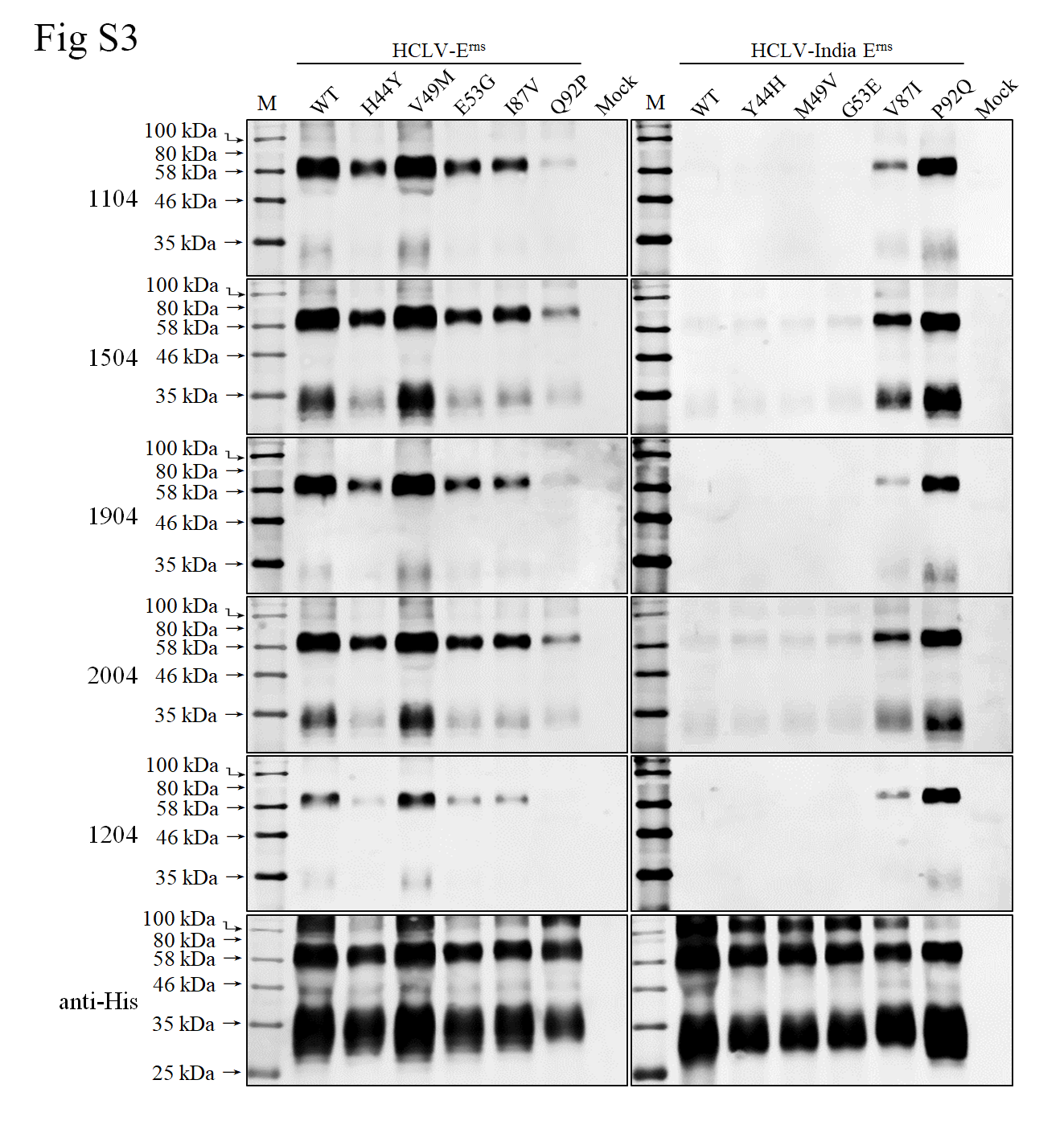

Supplement: Supplementary Figure 3 — Determination of the critical residues responsible for difference of Erns reactivity with 5 anti-Erns mAbs between Chinese HCLV and HCLV-India. Bottom panel is the normalization of mutated Erns proteins for each lane as detected by anti-His mAb. [file Image_3.tif]
