# Supplementary material for: Characterization of monoclonal antibodies that specifically differentiate field isolates from vaccine strains of classical swine fever virus
Source: Front Immunol. 2022 Jul 19;13:930631. doi: 10.3389/fimmu.2022.930631 (PMC9361847; doi:10.3389/fimmu.2022.930631)
Supplement: Supplementary file 4 [file Table_1.docx]

| Table S1. CSFV field isolates and vaccine strains of other sub-genotypes unavailable in our laboratory | | | | |
| --- | --- | --- | --- | --- |
| CSFV | CSFV strain name | Genotype | Genbank accession No. | Reference |
| Field strain | Brescia | 1.2 | AY578687 | (17) |
|  | CSF0650 | 1.3 | JX028200 | (7) |
|  | CSF1056 Holguin | 1.4 | JX028202 | (7) |
|  | CSF0410 Congenital Tremor | 3.1 | JQ411575 | (38) |
|  | JJ9811 | 3.2 | KF669877 | (39) |
|  | 94.4/IL/94/TWN | 3.4 | AY646427 | (40) |
| Vaccine strain | HCLV-India | 1.1 | EU857642 | (41) |
|  | GPE- | 1.1 | D49533 | (42) |
|  | LK-VNIVViM | 1.2 | KM522833 | (43) |
|  | LPC | 1.1 | AF352565 | (41) |
|  | Riems | 1.1 | U45477 | (44) |
|  | Rovac | 1.2 | KJ873238 | (43) |
|  | Thiverval | 1.1 | EU490425 | (44) |
